# Function Integration in Additive Manufacturing: Design and Realization of an LPBF Built Compressed Air Motor

**DOI:** 10.3390/ma15196632

**Published:** 2022-09-24

**Authors:** Benedikt Adelmann, Ralf Hellmann

**Affiliations:** Applied Laser and Photonics Group, University of Applied Sciences Aschaffenburg, Wuerzburger Strasse 45, 63739 Aschaffenburg, Germany

**Keywords:** selective laser melting, additive manufacturing, function integration, air motor

## Abstract

We present a compressed air motor, completely built by laser powder bed fusion. To highlight the fully functional integration by additive manufacturing, the rotor, stator, bearings, turbine, gas inlet and outlet were all built in a single print job. The material used was Inconel 718, and the motor was 44 mm tall and 12 mm in diameter. With the rotation axis of the motor in print in the direction of the laser powder bed fusion process, no build supports are needed, and thus the rotor and stator are fully moveable against each other. Plain bearings were used to position the rotor inseparably inside the stator, with a bearing gap of 0.2 mm, resulting in stable rotation. The idle rotation speed of the motor was measured with a digital laser measuring device as a function of air pressure and inlet size. With linear behavior between the rotation speed and gas pressure of up to 5.5 bar, the motor can be easily controlled. With varying gas inlet sizes, the idle rotation speed of the compressed air motor is also varied. A maximum rotation speed of 90,000 rpm was achieved at 1.5 mm gas inlet size and 3 bar gas pressure.

## 1. Introduction

While additive manufacturing (AM), in general, continues to gain considerable attention in both science and industry, function integration in particular forecasts one of the major opportunities for future applications of AM [1,2,3,4]. Beyond fundamental studies on material and parts properties, process optimization and process monitoring [5,6,7,8], the demonstration of exemplifying applications is of upmost importance to foster the implementation of the unique advantages of AM, as being the particular scope of this special journal issue. Among others, selective laser melting (SLM), also referred to as laser powder bed fusion (LPBF), has been shown to allow for the realization of dental frameworks with superior fitting accuracy [9], patient individualized osteosynthesis plates [10], lightweight structures [11], tools for die casting with integrated channels [12] and turbine blades [13].

Function integration is one of the key drivers for advanced AM, leading to fewer components and mounting efforts when producing complex systems, in turn resulting in higher economic viability and sustainable resource efficiency. In this respect, by facilitating new geometries, the use of AM yields reduced cooling air consumption and increased efficiency for gas turbines [14]. Two major challenges for function integration are the print direction in order to avoid unremovable support between movable parts and the clearance between movable parts to ensure movability only in the desired direction [15]. In detail, when a joint should only rotate, it must be ensured that only minimal tilt or dislocation movements are possible. Further challenges are to replace unproducible parts such as ball bearings with producible parts while retaining the function.

Specifically, using LPBF has led, for example, to a reduction in the number of components to be assembled for building the pedal system of a car, in turn resulting in a weight reduction of 24% as compared to conventional manufacturing [16]. This has also been used for improved tools for die casting with complex cooling channels allowing for shorter molding cycles and reduced surface finishing of the molded components [12]. Function integration using LPBF has been shown to create reactors with different inner contours to optimize the catalytic effects for the production of liquid fuels from carbon monoxide and carbon dioxide [17], or moveable operational components such as helical springs [18,19] or mechanical joints [15].

An intriguing example of function integration is exemplified by gears and motors. The possibility of building parts of an electromotor or turbines such as rotors or turbine blades by LPBF has recently been demonstrated by generating the complete rotor of a synchronous reluctance motor in one part [20]. A rotor for an electromotor, which additionally includes an impeller to produce an air flow, has also been produced by LPBF [21]. Using soft magnetic alloys for stators, LPBF has been used to build components of electric motors with low iron loss [22]. For turbines, LPBF has been used for several applications such as producing turbine blades [23] or repairing damaged blades [24], with a research focus on print quality in terms of grain microstructure, mechanical strength and high temperature creep resistance [25].

Against this background, in this contribution we design, additively build and characterize a fully functional, integrated compressed air motor. Taking advantage of the possibility to build channels for compressed air by LPBF [26,27,28], the motor can be supplied with energy at various positions.

## 2. Experimental Section

### 2.1. SLM Process

**Figure 1:**
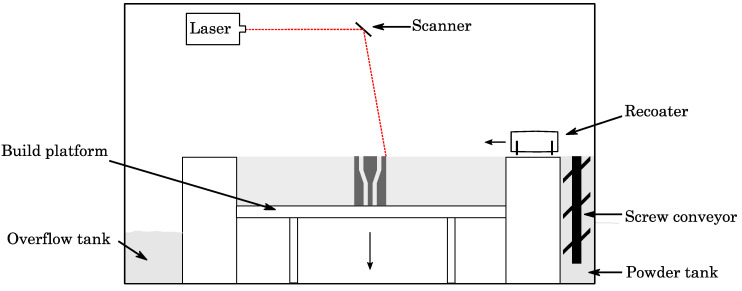
A scheme of the SLM machine.

For laser powder bed fusion, we employed a Realizer SLM 50 (DMG Mori, Bielefeld, Germany) equipped with a 100 W yttrium fiber laser (YLM-100; IPG Photonics, MA, USA). The spot size of the laser was set to d_spot_ = 20 μm at focus position. The machine processed under an argon atmosphere with a 0.1% oxygen level. The build platform was kept at 200 °C to maintain the part at an elevated temperature, to avoid deformation by curling due to residual stress. The machine parameters used included a layer thickness of 25 µm, hatch distance of 60 µm, laser power of 55 W and an exposure time of 20 µs. A schematic of the machine used is depicted in Figure 1. In the machine, the powder is transported by a screw conveyor from the powder tank towards the recoater. The recoater distributes the powder as a thin layer all over the build platform and surplus powder is dumped into an overflow tank. The powder on the build platform is locally melted and welded by a fiber laser to the underlying metal part. Then, the build platform moves downwards and the process starts again to perform the next layer until the part is built completely.

The powder used in the experiments was a Nickel Superalloy Inconel 718 (Heraeus, Hanau, Germany), a hardenable nickel–chromium-based alloy with a grain size between 15 µm and 45 µm. The chemical composition is shown in Table 1. The advantage of this alloy is its high creep rupture strength at high temperatures up to 700 °C and the high mechanical strength of >960 MPa in combination with a high corrosion resistance. The literature results of the SLM build samples report a mechanical strength between 878 MPa and 1070 MPa for Inconel 625 depending on the build direction, which reveals that the datasheet strengths are usually achieved in SLM build processes [29]. Typical applications of this alloy are its use in gas turbines, exhaust components, heat exchangers or aircraft engines.

### 2.2. Device Characterization

Geometrical deviations between the digitally designed and physically realized motors, i.e., the verification of shape accuracy, were determined by structured light scanning using an Atos Core 3D scanner and employing shape variance analysis using ATOS Professional V8 SR1 software (GOM GmbH, Braunschweig, Germany). For closer inspection of the inner parts of the fully integrated motor, optical images of metallurgical cross sections were taken by a Leica DVM6 digital microscope (Leica Microsystems GmbH, Wetzlar, Germany).

### 2.3. Design

**Figure 2:**
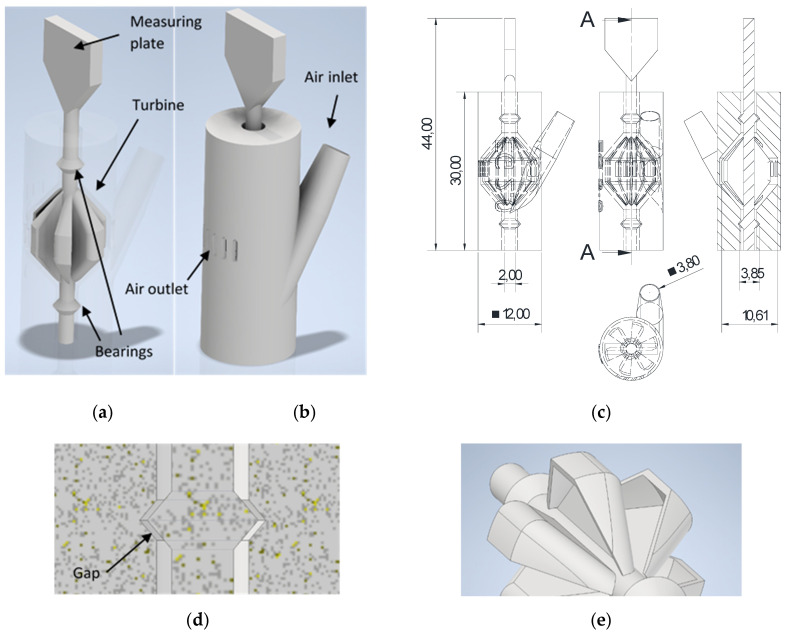
CAD images of the pneumatic motor with a view of the: (**a**) rotor; (**b**) exterior view; (**c**) dimensioned view with units in Millimeter; (**d**) bearing with a gap; and (**e**) cups of the turbine.

The design of the pneumatic motor is mainly influenced by the design rules of the LPBF process and is depicted in Figure 2. The most important design rules are the avoidance of overhangs larger than 40°, avoidance of large surfaces in the building layer, building with the least volume possible and circumventing support structures wherever possible. Because the function integrated motor should be built as one piece without the need to assemble different parts together, the rotor and stator are designed to be movable but inseparable from one another. To enable movability between the rotor and stator, the motor is built upright, i.e., the rotational axis is vertical. This avoids the need for support structures between the rotor and stator. In addition, the design is almost symmetrical, so the motor can also be built upside down which increases the integration flexibility. As depicted in Figure 2c, the motor has a diameter of 12 mm and a height of 30 mm, plus 14 mm for the measuring plate. The rotor shaft has a diameter of 2 mm and the turbine diameter is 10.61 mm. The complete weight of the motor is 25 g.

The only actual contact between the rotor and stator occurs in the bearings (Figure 2a). Because ball bearings or rolling bearings are not possible to produce without any support structures, which are not removable after the SLM process as they are inside, a simple plain bearing is used. The bearing consists of a double cone-shaped belling at the rotor and a V-shaped notch in the stator as seen in Figure 2d. The maximum diameter of the cone is larger than the minimum inner diameter of the housing. Due to this shape, the rotor is fixed in both the direction of the rotation axis and perpendicular to the rotation axis. Please note, the size of the gap between the rotor and stator was analyzed in detail in Section 3.2. However, the gap has to be smaller than the gap between the turbine and housing in order to avoid contact between the both of them and to reduce friction torque. The maximum angle of overhang was set to 40° in order to achieve high build quality, because higher overhang results in a high surface roughness and thermal distortion [30,31].

The turbine is designed as a tangential turbine, where the gas flow runs perpendicular to the rotation axis, similarly to the design of a cup anemometer. The use of an axial turbine was hampered by the maximum overhang angle of 40° of the turbine blades, resulting in a low power transmission of the gas to the turbine. The tangential turbine used has a tapered shape on the top and button (Figure 2a) in order to minimize the overhang angle. The turbine has 6 blades, on the fringes of which side bumps (see Figure 2e) are placed, giving the blade a cup shape to increase the air resistance in one direction.

The gas inlet is designed as an inclined pipe to reduce the overhang angle. On the top side, the outer surface is cone-shaped and was sized with an outer diameter of 3.8 mm to fit for a standard 8 mm compressed air hose. In an application, LPBF build channels can also be placed at this position [12]. Between the pipe and the turbine, a house-shaped gap was placed to reduce the cross section area and to guide the gas flow in a horizontal direction onto the turbine. The air outlet was placed on the opposite side of the air inlet, consisting of five upstanding rectangular breakthroughs. On the top of the breakthroughs, a roof geometry avoids high overhang angles. Due to the multiple air outlets, pressure can be reduced slowly and airflow is better utilized.

### 2.4. Rotation Speed Measurement

After the LPBF process, the engine was detached from the building platform. The engine was further freed from powder residues and flushed with oil. This has the advantage that the last powder residues are removed from the interior, which could impair the smooth running of the engine. The engine was lubricated by the oil for a smoother and damage-free run. Different types of oil were tested for this. The oil type “Wet Lube” from “Muc-Off”, consisting of a mixture of synthetic-based oils with additives, turned out to be the most suitable, leading to the highest rotations speeds without perturbing fluctuations.

**Figure 3:**
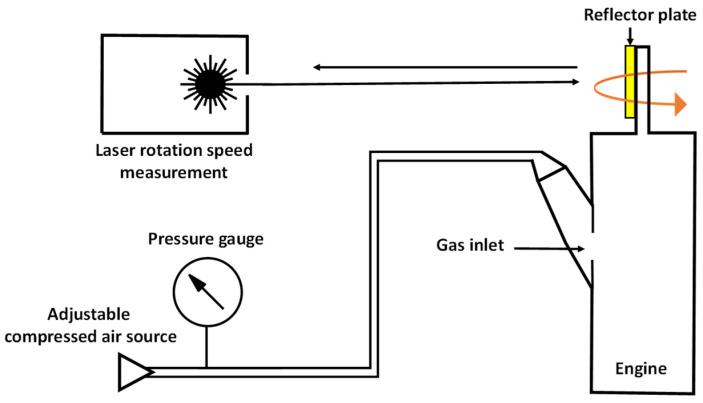
The experimental setup of the rotation speed measurement.

Rotation speed measurements were performed using a commercial digital laser handheld measuring device from KS TOOLS (model 455.0130, KS TOOLS GmbH, Heusenstamm, Germany) as depicted in Figure 3. The maximum measurable rotation speed of this device is 100,000 rpm and the display is actualized once per second. For a better performance, reflective tape was affixed to the measuring plate on the rotor. For a more precise measurement, the back side of the rotor was colored dark, which increased the contrast with the bright reflector plate. When the laser hits the side of the rotor with the reflective tape, the laser beam is reflected towards a photodiode in the measuring device with the opposing blackened side being non-reflective. The measuring device calculates the rotation speed based on the number of reflections per time interval.

Both the engine and the measuring device were statically fixed in order to ensure a reproducible measurement setup. The measurement device was directed to the reflector plate by a guide laser. The engine was supplied with compressed air via an 8 mm hose and the inlet gas pressure was measured with a pressure gauge. For the measurements, the compressed air was switched on and adjusted to the desired pressure. After waiting for two seconds to ensure that the pressure reached the engine and the engine reached a stable rotation speed, the median value of the five following displayed rotation speeds on the measuring device was used.

## 3. Results

### 3.1. Part Characterization

**Figure 4:**
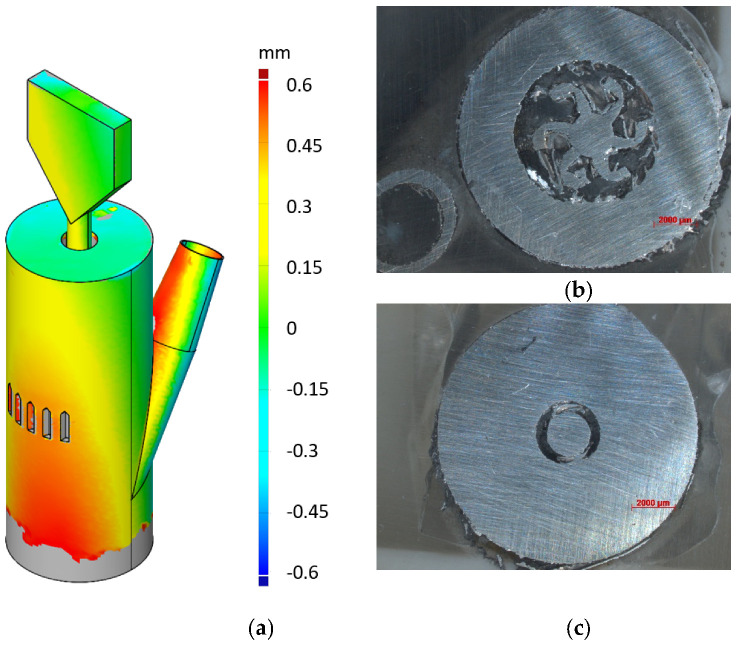
(**a**) An optical 3D scan and false color representation of the LPBF built device showing shape and dimensional deviations. Cross section microscope images of (**b**) the turbine and (**c**) the shaft.

To basically characterize the LPBF built compressed air motor, both optical 3D scans of the entire device as well as cross section images at different component heights were taken. A typical 3D scan of the motor is shown in Figure 4a, revealing the deviation between the surface of the 3D scan and the desired surface from CAD data in a false color representation. In this representation, the color red indicates additional material, while the color blue indicates a lack of material. Please note, grey coloring at the bottom of the device is caused by the fixation of the motor on the base plate. The gas inlet is indicated at a higher position than expected because of the red color at the top side. The continuous color gradient from the bottom to the top of the motor can be assigned to a slight misfit of the fitting algorithm between the 3D scan and CAD data. Nevertheless, it is noteworthy that no significant thermal distortion or bending of the shaft was detected. To gain further insight into the interior of the built motor, cross section images were taken by embedding the motor into plastics and sawing in a horizontal plane, i.e., perpendicular to the rotation axis. The surface was further ground to achieve a flat surface before examining the surface under the microscope. In Figure 4b, an exemplarily chosen cross section of the turbine section is given, clearly revealing the cup shape of the turbine as designed in the CAD data. In addition, the turbine is apparently fully centered in the cavity and the turbine blades do not touch the cavity walls except for a slight connection on the right side. This connection to the wall is based on a powder particle or a splinter from sawing. Furthermore, Figure 4c depicts a cross section at the height below the turbine. Again, the shaft is well centered in the cavity without contact with the sidewalls, proving that the inner contours are well built as designed.

**Figure 5:**
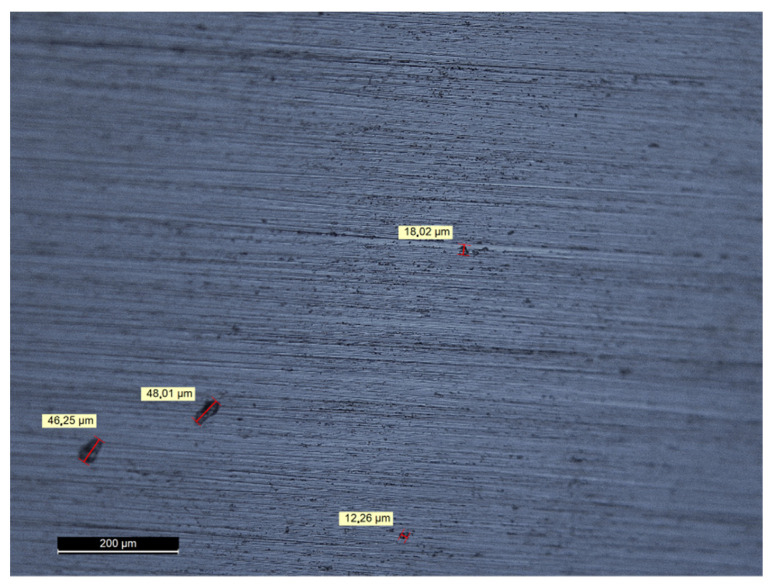
A cross section image of printed Inconel to show the pore distribution.

To provide a closer look into the material in order to determine pore behavior, a micrograph of the cross section is shown in Figure 5. Here, various sized pores are visible with a maximum size in the range of 50 µm, but such large pores were quite low in number. More often, pores with sizes between 10 µm and 20 µm were found. This is not unusual because literature results reveal the porosity of Inconel as build samples, depending on the laser parameter combination, between 0.8% and 3%, with a typical pore size of 10 µm to 40 µm, and with maximum pore sizes of 200 µm beyond the material surface [29].

### 3.2. Bearing Gap Distance

Initial tests aimed to investigate which distance in the bearing between the rotor and stator is necessary to enable the rotor to rotate and simultaneously ensure a stable rotor position. The rotor bearing is designed with an overhang, while in LPBF processes, the fabrication of overhangs, in general, leads to a change in the surface roughness. Fox et al. report on changes of the surface roughness R_a_ of 20 µm to 35 µm when the angle of the overhang increases from 15° to 60° [30]. In LPBF, various factors lead to shape and dimensional deviations between the CAD-constructed part and the actual build part, such as, process parameters including laser beam diameter and applied laser power [6,32,33], adhering powder particles or thermal deformations [29]. Fundamentally, the digital process chain may also lead to dimensional deviations [34]. Therefore a deviation of 0.1 mm is not atypical [35]. In addition, bearing gap cannot be measured due to the special closed housing design of our pressure air motor. As a consequence, the necessary distance between the rotor and stator was determined experimentally. For this purpose, different distances on each side between 0.05 mm and 0.3 mm in steps with a size of 0.05 mm were examined. The parameter study revealed that a distance of 0.2 mm is the best, because motors with higher or lower distances revealed no continuous rotation under feeding with pressured air. This ensured a clean rotation of the motor when air pressure was applied. With smaller distances, especially 0.1 mm and less, the rotor could hardly be turned by hand. A reason for this could be the high roughness, which interlocks the stator with the rotor, or the cant of particles in the gaps. The used powder had a maximum grain size of 45 µm, but during the SLM process grains can be welded together which results in larger grains. Such grains may cant between rotor and stator and being fixed there due to the high roughness.

With larger gaps, the rotor sticks because of the high room to move freely, so the turbine hits the surrounding housing when it rotates. Due to the higher diameter of the turbine as compared to the bearing, the same friction force results in a higher braking torque, which can exceed the torque generated by the air pressure, resulting in no rotation. In the literature, gaps between 0.2 mm and 0.5 mm are found to be optimal which agrees with our determined gap [15]. As a result, a bearing distance of 0.2 mm provided a good compromise between a high enough rotation movability of the rotor and a good position fixture and was therefore used in the following tests.

### 3.3. Dependence on Gas Pressure

**Figure 6:**
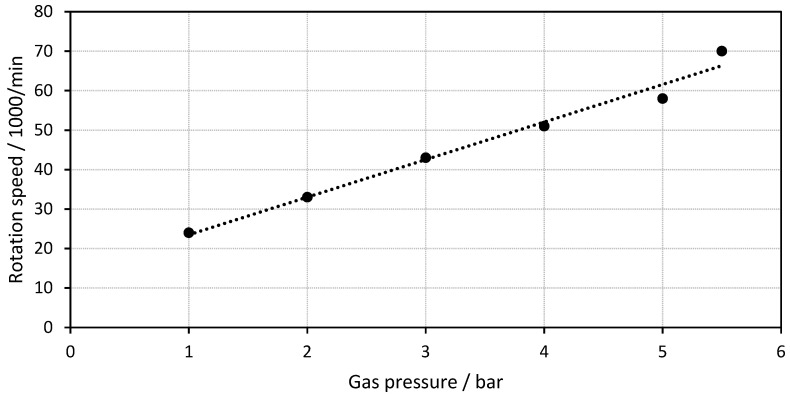
Rotation speed as a function of the gas pressure.

As a key performance characteristic, idle rotation speed is measured as a function of applied air pressure for the optimized motor. The measured rotation speed for a motor with 1 mm gas inlet size as a function of the air pressure is shown in Figure 6. The rotation speed increased linearly with air pressure from 24,000 rpm at 1 bar to 70,000 rpm at 5.5 bar. This increase in rotation speed with the gas pressure is typical for air motors [36]. The linear behavior simplifies the control of the motor and makes it flexible due to the wide range of rotation speed.

This rotation speed is in the range of typical industrial compressed air motors with small sizes, using turbine, lamella or gear wheel designs, which have maximum rotation speeds between 60,000 rpm and 120,000 rpm. Small drilling and grinding machines also use rotation speeds in ranges up to 80,000 rpm, while dental drilling machines usually have higher rotation speeds in the area of 200,000 rpm. The overall performance of our build motor is lower as compared to standard air motors in terms of power density and torque due to missing parts such as complex ball bearings, rubber seals and the lower accuracy due to the high roughness. Nevertheless, our motor was produced in one production step within one machine and can be integrated into larger SLM parts, which is the main advantage of our motor design.

### 3.4. Gas Inlet Size

**Figure 7:**
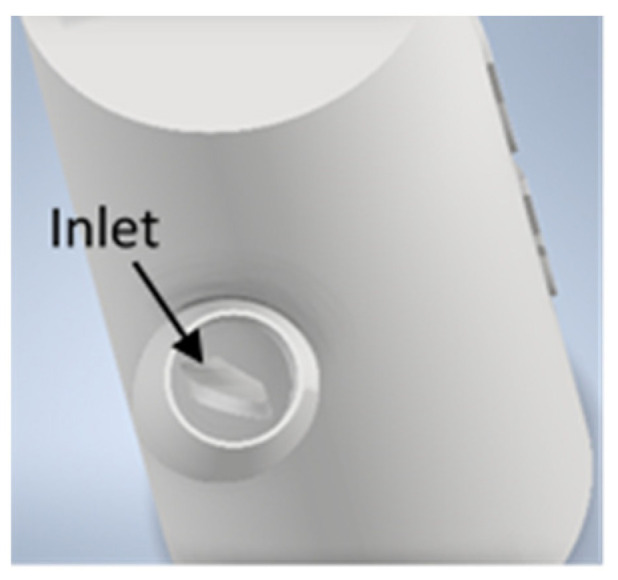
A CAD image of the gas inlet.

To further improve the compressed air motor, the gas inlet size was varied in order to produce motors with high idle running speeds. Therefore, the house-shaped inlet size as depicted in Figure 7 is varied in width between 0.5 mm and 2.5 mm, in 0.5 mm incremental steps.

**Figure 8:**
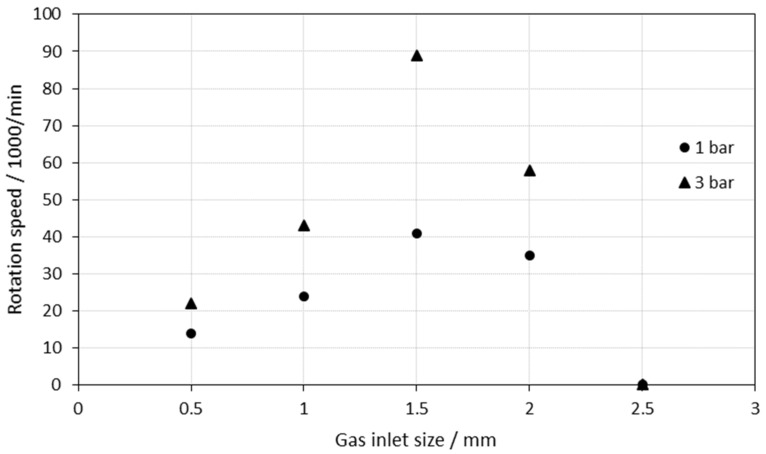
Rotation speed as a function of the gas inlet size.

The resulting rotation speed for different gas inlet sizes with a gas pressure of 1 bar and 3 bar is depicted in Figure 8. For both gas pressures, the rotation speed increases up to a gas inlet size of 1.5 mm. This can be assigned to the higher gas flow rate, transmitting a higher momentum to the turbine. With a maximum rotation speed of 90,000 rpm at 3 bar and 1.5 mm inlet size, this motor is attractive for applications such as small milling machines or dental drilling. With further increasing gas inlet sizes, the rotation speed decreases and at 2.5 mm inlet size, the motor does not move at all. We attribute this to the high gas pressure in the complete turbine chamber, which hampers a directional gas stream onto the turbine blades, i.e., an increase in the gas inlet size does not necessarily lead to an increase in the maximum speed. In conclusion, for the constructed compressed air motor, a gas inlet size of 1.5 mm reveals the highest speed for both 1 bar and 3 bar air pressure.

## 4. Conclusion

Exemplifying fully functional integration in laser powder bed fusion, we have demonstrated the design, realization and characterization of a compressed air motor with a diameter of 12 mm and height of 44 mm, completely built within one additive manufacturing print job using Inconel 718. The motor consists of bearings, a turbine, gas inlet and outlet as well as a plate for rotation speed measurements. The rotor and stator are printed moveable, yet inseparable together with the rotation axis in the print direction in order to avoid support structures. Optical 3D scanning using structured light scanning and cross-sectional images revealed superior geometrical accuracy. The motors were characterized by measuring their idle rotation speed as a function of different bearing gaps, gas inlet size and gas pressure. A bearing gap of 0.2 mm, and the initial cleaning and flushing of the motor with oil is necessary to achieve continuous rotation. Rotation speed as a function of the gas pressure shows a linear characteristic as being advantageous for motor control. With different gas inlet sizes, the motor revealed a maximum rotation speed of 90,000 rpm at 1.5 mm gas inlet size and 3 bar. The results highlight the possibility to produce the fully functional integration of compressed air motors within one laser powder bed fusion build job.

## Figures and Tables

**Table 1:** The chemical composition in wt.% of the Inconel 718 superalloy.

Ni	Cr	Fe	Nb	Mo	Ti	Al
50–55	17–21	Balance	4.7–5.5	2.8–3.3	0.65–1.15	0.2–0.8

## Data Availability

All Data used in the manuscript is presented in the diagrams. There is no further data available.
